# Bioinformatics analyses of a potential miRNA‒mRNA regulatory axis in lumbar degenerative disc disease

**DOI:** 10.1186/s12920-026-02372-z

**Published:** 2026-04-21

**Authors:** Jiagang Wang, Lifan Zhu, Fengbiao Weng, Jincai Zeng, Liang Xu, Yuwei Chen, Yuhui Shi

**Affiliations:** Department of Orthopedics, Suzhou Ninth Hospital Affiliated to Soochow University, No. 2666 Ludang Road, Taihu New Town, Wujiang District, Suzhou, Jiangsu 215200 China

**Keywords:** Intervertebral disc degeneration, Nucleus pulposus, miRNA, CPNE6, B3GAT1

## Abstract

**Background:**

Intervertebral disc degeneration (IDD), normally characterized by a loss of nucleus pulposus cells (NPCs), is one of the leading causes of lower back pain and various degenerative spinal disorders and has been regarded as a public health issue because of its heavy social and economic consequences. However, the molecular mechanisms underlying IDD formation and progression are unclear so far, which results in the lack of existing biomarkers or treatments in targeting early degeneration effectively. Novel potential biomarkers for the early diagnosis, prevention and treatment of IDD are urgently needed.

**Methods:**

In this study, miRNA sequencing and qRT-PCR validation were performed on original clinical nucleus pulposus (NP) tissues from our own IDD patient cohort. Furthermore, we applied bioinformatics analysis to the mRNA expression profiles in NP tissues (GSE186542) and whole blood (GSE124272) from IDD patients. We screened key genes and miRNA regulators by conducting overlap analysis.

**Results:**

The results showed that 466 differentially expressed miRNAs, 187 downregulated and 279 upregulated significantly, were identified from miRNA sequencing analysis in NP tissues. Overlap analysis with the predicted miRNA targets and the differentially expressed genes (DEGs) in the GSE186542 database exhibited 27 overlapping genes, among which copine-6 (CPNE6) and beta-1,3-glucuronyltransferase (B3GAT1) overlapped with the DEGs from the whole blood of IDD patients in the GSE124272 database. Further qRT‒PCR results revealed that CPNE6 and B3GAT1 expression was significantly upregulated, but their corresponding miRNA regulators miR-3620-5p and miR-6511b-3p were significantly downregulated in IDD patients. Moreover, cells proliferation was inhibited, but the IL-1β, IL-18 and TNF-α contents were significantly increased, in ATDC5 cells after miR-3620-5p and miR-6511b-3p inhibition.

**Conclusion:**

Thus, miR-3620-5p and miR-6511b-3p might be the key effectors in IDD progression by mediating the expression of CPNE6 and B3GAT1 in NP tissues. This newly discovered specific miRNA‑mRNA interactions were identified and validated using our original patient samples, with public datasets used for bioinformatic cross‑validation. It holds huge potential applications for miRNA-based therapies in early diagnosis and treatment of IDD.

**Supplementary Information:**

The online version contains supplementary material available at 10.1186/s12920-026-02372-z.

## Background

IDD is a complex phenomenon, contributing a main cause of low back pain (LBP), which imposes an increasingly substantial financial burden on families and society [1–3]. Various factors can cause IDD, such as aging, heredity, mechanical stress, obesity, and even smoking. The IDD occurs as early as adolescence, and with the age increases, the population affected by IVD increases sharply. According to the statistics, about 10% of the 50-year-old population suffers from IDD, while it is about 60% in the 70-year-old population (PMID: 35486489). Conventionally, treatment strategies for IDD mainly focus on alleviating clinical symptoms, including surgical and pharmacological interventions [4]. Recent advancements proposed that nanocarrier drug delivery systems (NDDSs), which designed for delivering various genes, cells and therapeutic drugs to target areas increased the precise and efficiency of IDD treatments (PMID: 38567254). It was newly proposed that surgical combination (MIS screw-rod for indirect decompression + interspinous fusion for long term spinal stability technique) exhibited excellent biomechanical stability (PMID: 38364897). In addition to efforts in current drug development and surgical strategies, considerable efforts have been made to explore the cause of its initiation and progression [3, 5], identifying novel genetic regulation for early diagnoses and treatment in IDD is still urgently needed.

Intervertebral discs (IVDs) are pads of fibrocartilage that resist compression by spreading loading evenly on the spinal bodies [6, 7]. The IVD is composed of the NP, peripheral fibrous anulus, and hyaline cartilage endplates [6]. NPCs mainly synthesize proteoglycan and fine collagen type II, and proteoglycan and water gel in the NP hold together loosely by elastin fibres and collagen type II, which provides the IVD with high strength to respond to hydrostatic pressure [8, 9]. IDD is characterized by a loss of NPCs and their replacement by cells with a fibroblast-like phenotype (PMID: 33557287). Therefore, external pathological conditions often influence the biological function of NPCs such that the extracellular matrix (ECM) synthesis ability decreases, which causes a series of pathological events, such as NP degeneration, IVD mechanical instability and rupture of the fibrous anulus, ultimately leading to corresponding clinical IDD symptoms. Recently, early intervention by promoting the repair and regeneration of the ECM to prevent IDD has gradually become popular. However, molecular markers for early diagnosis are in high demand and still lacking.

miRNAs are small noncoding RNAs that bind to specific mRNA targets and posttranscriptionally regulate target gene expression and participate in various physiological and pathological processes [10–12]. Emerging evidence shows that miRNAs are associated with the pathogenesis of most kinds of cancer and angiogenesis [12–14]. Recent studies have confirmed that miRNAs may play crucial roles in IDD progression by regulating NPC apoptosis and proliferation, inflammation, ECM degradation and/or annulus fibrosus (AF) degeneration [15, 16]. For example, miR-19b-3p was suggested to inhibit human NPC apoptosis in IDD via involvement in PTEN/PI3K/Akt/mTOR signalling [17]. miR-141-3p activated the Keap1-Nrf2 pathway to attenuate IDD symptoms in mice [18]. A study from patients with IDD and idiopathic scoliosis indicated that miR-27a-3p actually induced the M1 polarization of macrophages and exacerbated IDD development (PMID: 37667246). miR-22-3p was confirmed to regulate IDD in mice by targeting SIRT, and inhibitions of miR-22-3p in NPCs exerted therapeutic roles (PMID: 37625164). Inflammatory responses are known as major events that occur during IDD. (PMID: 33557287). Recent studies indicated that miRNA plays crucial roles in the pathogenesis of inflammatory diseases and regulating various cell biology such as cell proliferation, invasion and migration. (PMID: 22211762, PMID: 23860189, PMID: 26805687). For example, miR-2355-5p was demonstrated to inhibit ERRFI1 expression, thereby mediating the proliferation and inflammation of NPCs in IDD. (PMID: 30817728) Considering miRNAs are highly conserved and tissue specific, specific miRNA profiling can distinguish diseases of different tissues, thus, miRNA-based therapy for IDD treatment is increasingly attractive [19, 20]. The design of miRNA-specific drugs and treatments for IDD heavily rely on patient evidences, especially molecular regulation underlying disease progression. However, due to the lack of miRNA targets for early-stage diagnosis or regenerative therapies, miRNA-based diagnosis or therapy makes tardy progress. Thus, more miRNA participants and their miRNA-mRNA regulatory mechanism in IDD patients are urgent need to reveal.

To uncover more miRNA mediators and their potential targets in IDD progression, we performed miRNA sequencing with NP samples from IDD patients and predicted miRNA target genes in this study. Furthermore, we applied bioinformatics analysis to the mRNA sequencing data in NP tissues (GSE186542) and whole blood (GSE124272) from IDD patients. After conducting overlap analysis step by step, we suggested that CPNE6 and B3GAT1 and their corresponding miRNA mediators miR-3620-5p and miR-6511b-3p might be the key regulators in IDD progression. Therefore, this study may provide a novel molecular mechanism of IDD and prospects for gene therapeutic strategies for IDD treatment.

## Materials and methods

### Patients

The NP tissues used in this experiment were collected from tissue specimens of The Ninth People’s Hospital of Suzhou in 2020 and diagnosed by two physicians separately; the final grading result was determined through consultation. Magnetic resonance imaging (MRI) diagnosis was performed before surgery, and the degeneration of the lumbar intervertebral disc was assessed on conventional T (2)-weighted images (T2WI) based on the Pfirrmann classification system. NP tissues with advanced stage degeneration (grade IV-V) [21] were used in this study and compared to normal tissues (Supplemental Table 1).

All miRNA sequencing and qRT-PCR experiments in this study were conducted using these original, prospectively collected human NP tissues from our own clinical cohort. Public gene expression databases (GSE186542 and GSE124272) were used exclusively for bioinformatic screening and cross-validation and did not serve as primary experimental data.

The research received written authorization from the participating patients after they were fully informed, and The Ninth People’s Hospital of Suzhou’s ethical boards granted their approval for the study.

### miRNA sequencing

Tissue samples from the human nucleus pulposus, obtained during spinal operations, were promptly preserved by immersion in liquid nitrogen. To assess the RNA levels, a Qubit^®^ RNA Assay Kit was employed within a Qubit^®^ 2.0 instrument for initial quantification, subsequent to which the samples were adjusted to a dilution of 1 ng/µl. The Agilent Bioanalyzer 2100 apparatus from Agilent Technologies in California, USA, was employed for the evaluation of insert size. Upon confirmation that the insert size met anticipated parameters, its precise quantification was accomplished through the employment of a Taqman fluorescent probe within the AB Step One Plus Real-Time PCR setup, ensuring a library concentration exceeding 2 nM. Sequencing of these approved libraries took place on an Illumina system, producing single-end reads of 50 base pairs in length. Sequencing depth was 10 million (10 M) reads [22].

### miRNA alignment and identification

The construction of the reference genome index employed Bowtie1, following which the purified sequence data were aligned to the genomic sequence. This alignment involved recording data for mature miRNA and hairpin structures in the miRBase database (version 21) to pinpoint established miRNAs. Subsequent to filtering out the sequences aligned with recognized miRNA, non-coding RNA, repetitive, and mRNA regions, the residual sequences served as the basis for the extrapolation of new miRNAs, with the prediction hinging on the hairpin’s form and its structural integrity. The tool utilized for both detection and prognostication of miRNAs was miRDeep2 [23].

### Differential expression analysis

DEGseq was used for differential gene expression analysis between two samples with nonbiological replicates, while DESeq was used for samples with biological replicates. Under the assumption that the number of reads derived from a miRNA follows a binomial distribution, DEGseq is proposed based on MA-plot and widely used for differential gene expression analysis. The P value could be assigned to each gene and adjusted by Benjamini and Hochberg’s approach for controlling the false discovery rate. miRNAs with q < 0.05 and |log2_ratio|≥1 were identified as differentially expressed miRNAs.

### miRNA target prediction

For animals, miRanda, PITA and TargetScan were used to predict target genes of known or novel miRNAs, with at least two software intersections [24]. miRanda is renowned for its comprehensive algorithm that takes into account multiple factors such as sequence complementarity between miRNA and target mRNA. TargetScan, on the other hand, focuses on conserved miRNA binding sites.

### Microarray data resources

The microarray data of GSE186542 comparing mRNA expression profiles of degenerative NP tissues to human control NP samples were obtained from the Gene Expression Omnibus (GEO) database (https://www.ncbi.nlm.nih.gov/geo/) (Supplemental Table 2). The mRNA expression profiles of the whole blood of patients with lumbar disc prolapse were obtained in the microarray data of GSE124272 compared to the tissues without clinical evidence of low back pain or sciatica (Supplemental Table 3). These data were applied for DEG identification, Gene Ontology (GO) and Kyoto Encyclopedia of Genes and Genomes (KEGG) enrichment analyses, and exploration of key genes overlapping in different subsets.

### Functional and pathway enrichment analyses

The GO (Gene Ontology, http://geneontology.org/) enrichment of miRNA target genes was implemented by the hypergeometric test, in which the p value was calculated and adjusted as the q-value, and the data background included genes in the whole genome. GO terms with q < 0.05 were considered to be significantly enriched. GO enrichment analysis revealed the biological functions of the target genes. Furthermore, the Kyoto Encyclopedia of Genes and Genomes (KEGG, http://www.kegg.jp) was referenced in the study. KEGG, a compendium of hand-illustrated pathways representing molecular interaction and reaction networks, utilizes the hypergeometric test for the enrichment analysis of target genes. This approach corrects for multiple testing by converting p-values to q-values. KEGG terms that demonstrated a q-value of less than 0.05 were deemed to have achieved noteworthy enrichment [25].

### Protein–protein interaction (PPI) network

The PPI network was constructed by the STRING database with CPNE6 and B3GAT1 selected as input (Version 11.0; www.string-db.org). A PPI score (medium confidence) ≥ 0.4 was employed as the cut-off value.

### qRT‒PCR

Total RNA was isolated from the NP specimens utilizing the TRIzol reagent provided by Invitrogen (based in the US), adhering to the protocol specified by the supplier. The conversion of this RNA into cDNA was achieved using the PrimeScript^®^ RT Master Mix Perfect Real Time system supplied by TAKARA (originating from Japan); this was followed by qPCR amplification with the aid of SYBR Green Master Mix, sourced from Applied Biosystems (US), employing the Applied Biosystems 7900HT Real-Time System for detection. To quantify relative gene expression levels, the 2^−ΔΔCt^ method was employed with normalization against the actin gene.

### Cell culture and treatment

The ATDC5 cell line was acquired from the American Type Culture Collection in Maryland, United States.

ATDC5 is a well-established murine chondrogenic cell line that mimics the biological characteristics of nucleus pulposus cells and cartilage endplate cells, and is widely used in IDD research due to its stability and reproducibility. Primary human NPCs are difficult to obtain in sufficient quantity, show high donor variability, and are challenging to transfect efficiently; therefore, ATDC5 cells were chosen for functional mechanistic assays.

These cells underwent cultivation in a mixture of DMEM/F12 nutrient medium (sourced from Thermo, USA) that was enriched with a 10% concentration of fetal bovine serum (FBS, also from Thermo, USA), and they were maintained in a moist incubator at a temperature of 37 degrees Celsius and a carbon dioxide level of 5%. In addition, ATDC5 cells were treated in 1% FBS (Thermo, USA) to induce cell degeneration.

### Cell transfection

The expression plasmids of miR-3620-5p and miR-6511b-3p inhibitor and NC were purchased from GenePharma Co. Ltd (Suzhou, China). They were transfected into ATDC5 cells using Lipofectamine 2000 Transfection Reagent (Thermo, USA) in accordance with the user’s protocol [26].

### Cell proliferation assay

We used cell counting kit-8 (CCK-8, MCE, USA) to measure proliferation. Firstly, cells were added to 96-well plates, followed by adding 10 µL of CCK-8 solution and incubating for 1 h. Then the absorbance at 450 nm was determined with Microplate Reader (BioTek, USA).

### Statistical analyses

qRT‑PCR experimental data were expressed as the means ± standard deviations (SD) and were analysed by GraphPad Prism 5.0 (GraphPad Inc., US) and SPSS 22.0 (IBM Corporation, US). To assess variance among distinct cohorts, we employed an Independent Student’s t-test or a one-way ANOVA. A P-value below 0.05 was deemed to indicate statistical significance.

## Results

### Predicting the targets of miRNAs differentially expressed in NP tissues of IDD patients

**Fig. 1:**
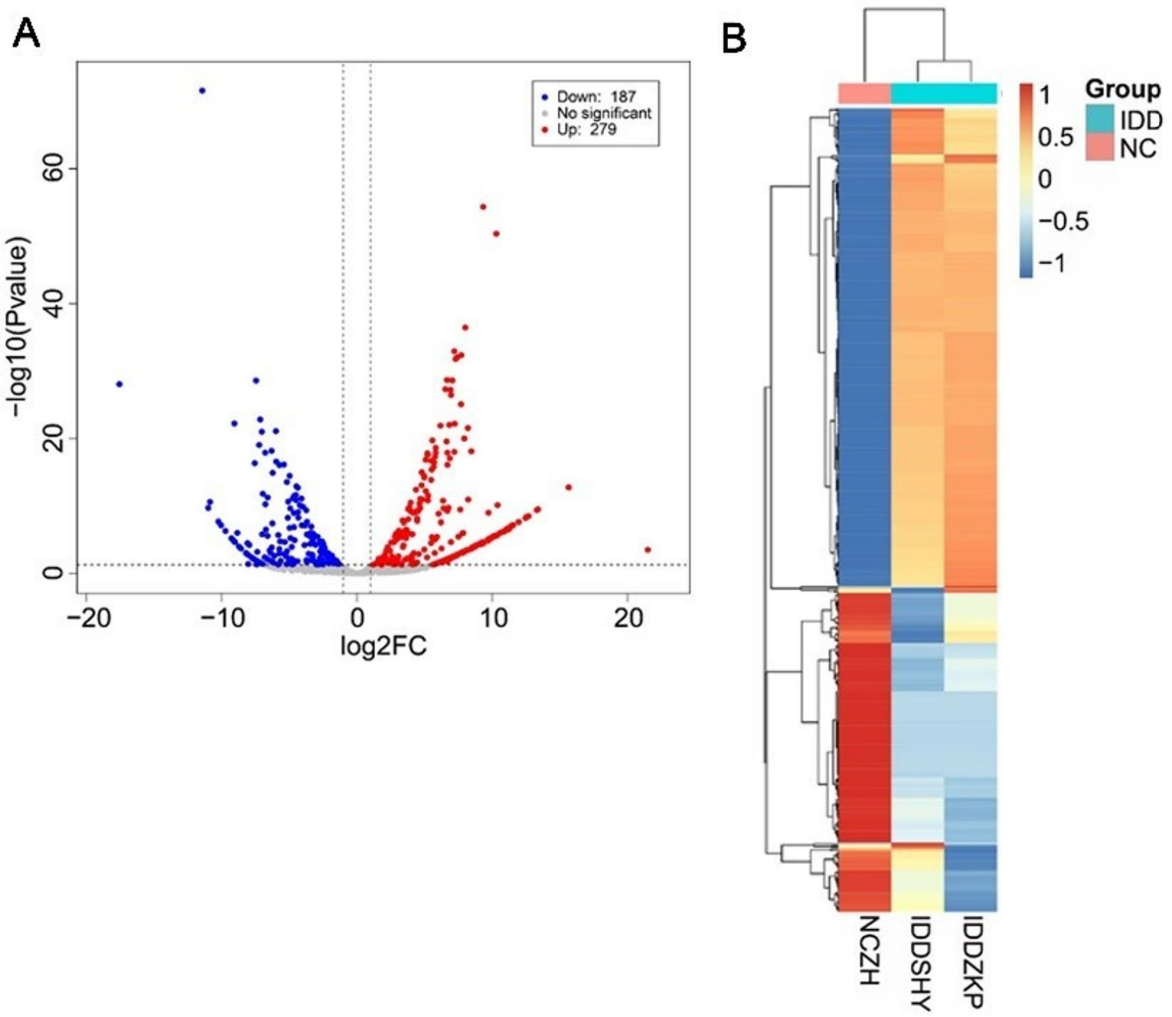
Volcano plot and heatmap analysis of the miRNAs differentially expressed in NP tissues of IDD patients. (**A**) Volcano plot showing 279 significantly upregulated and 187 significantly downregulated miRNAs. The x-axis is log2 (fold change), and the y-axis is -log10 (*P* value). (**B**) Heatmap exhibits the expression of these miRNAs. Each column represents one dataset, and each row represents one gene. NCZH represents the normal control sample, and IDDSHY and IDDZKP represent two IDD samples. The gradual colour change ranging from blue (-1) to red (1) represents the changing expression from downregulation to upregulation

To explore key molecular mediators in IDD progression, we first performed de novo miRNA sequencing on our own original NP tissues from IDD patients and controls (Supplemental Table 4). The volcano plot results showed 187 miRNAs that were significantly downregulated and 279 miRNAs that were upregulated under the criteria of |log2 FC| > 1 and *p* value < 0.05 (Fig. 1A). The heatmap analysis revealed the detailed expression and distribution status of miRNA expression profiles (Fig. 1B).

**Fig. 2:**
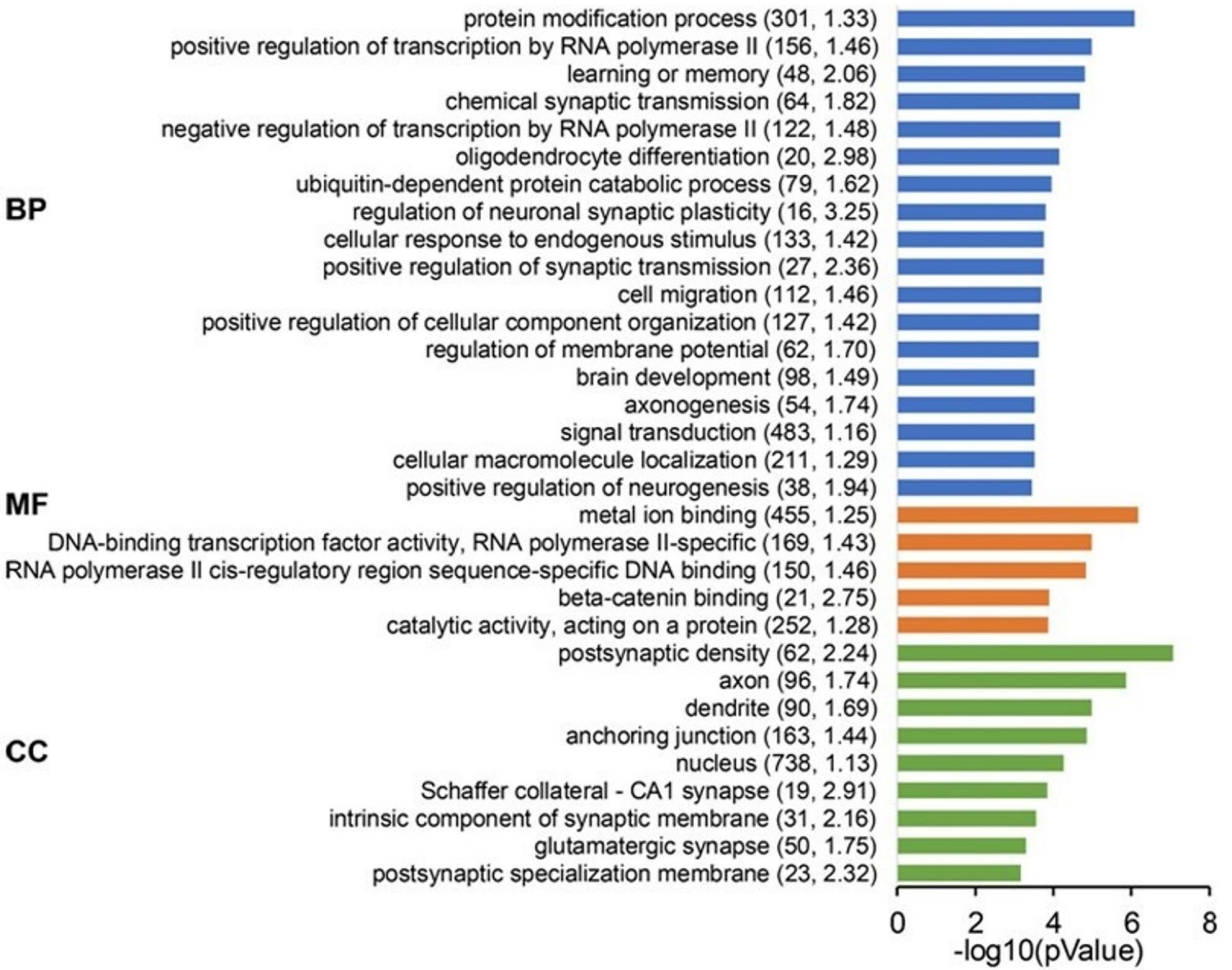
GO analysis of the predicted miRNA targets. GO enrichment analysis of the top 32 enriched significant GO terms classified as biological process (BP, blue), cellular component (CC, green), and molecular function (MF, orange). The x-axis was -log10 (*P* value), and the y-axis was the name of the GO terms

**Fig. 3:**
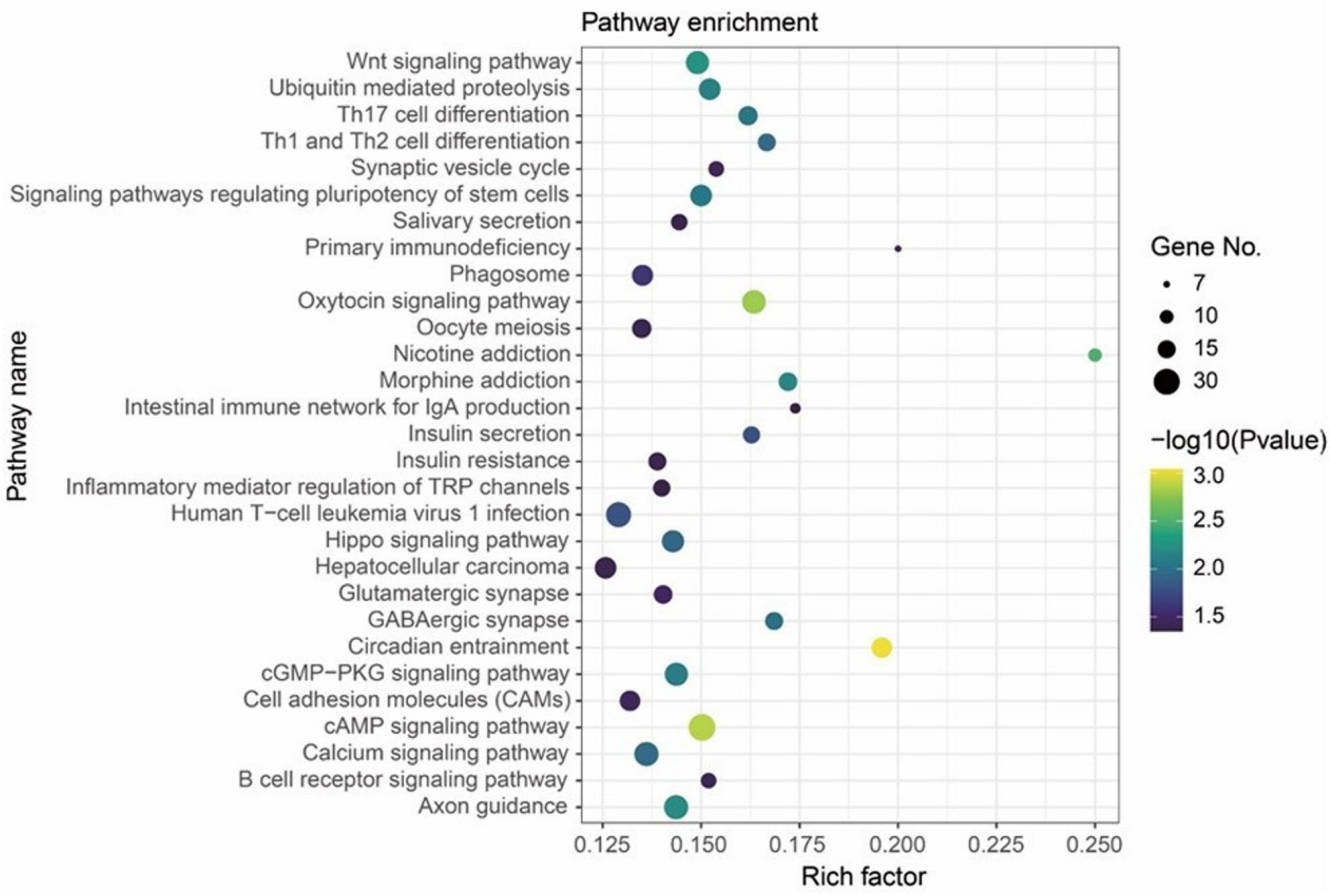
KEGG analysis showing the top 30 enriched signalling pathways of the predicted miRNA targets. The x-axis is the rich factor, the size of the dots represents the number of genes, and the gradual colour change ranging from blue (5) to yellow (12.5) represents the score of -log10 (*P* value). The y-axis shows the name of the enriched signalling pathways

Moreover, we predicted the targets of these miRNAs with altered expression in NP tissues of IDD patients via the online databases miRDB and MirTarget2 (Supplemental Table 4). We performed GO and KEGG analyses to identify the functional and signalling pathway enrichment of these explored mRNA targets. The top 32 significantly enriched GO terms were classified into biological process (BP), cellular component (CC) and molecular function (MF), and the terms in BP included cell migration, axonogenesis, regulation of neuronal synaptic plasticity, and positive regulation of cellular component organization, which are cell activities closely associated with IDD progression (Fig. 2 and Supplemental Table 5). Furthermore, we found that the significantly enriched KEGG pathways of the mRNA targets included the cAMP signaling pathway and Wnt signaling pathway (Fig. 3 and Supplemental Table 6).

### Identifying the DEGs in NP tissues of IDD patients via dataset GSE186542

**Fig. 4:**
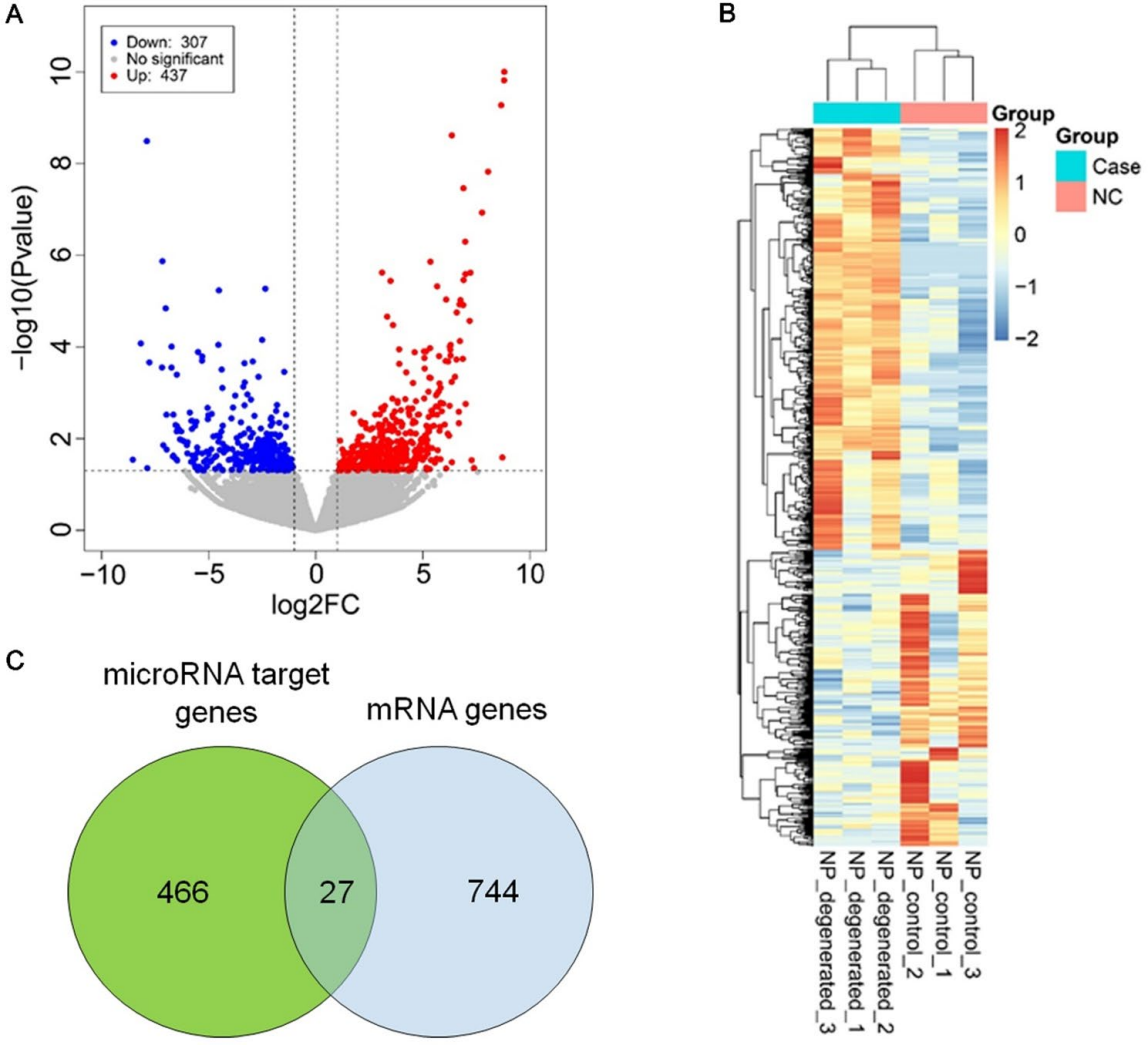
The volcano plot and heatmap of DEGs from the expression profile of degenerative NP tissues in dataset GSE186542. (**A**) Volcano plot showing 437 significantly upregulated and 307 downregulated mRNAs. The x-axis is log2 (fold change), and the y-axis is -log10 (*P* value). (**B**) Heatmap of the DEGs from 3 degenerative NP samples compared to 3 controls. Each column represents one tissue sample, and each row represents one gene. The gradual colour change ranging from blue (-2) to red (2) represents the changing expression from downregulation to upregulation. (**C**) Overlap analysis of the 466 predicted miRNA targets and 744 significant DEGs from dataset GSE186542. Twenty-nine overlapping genes were identified

**Fig. 5:**
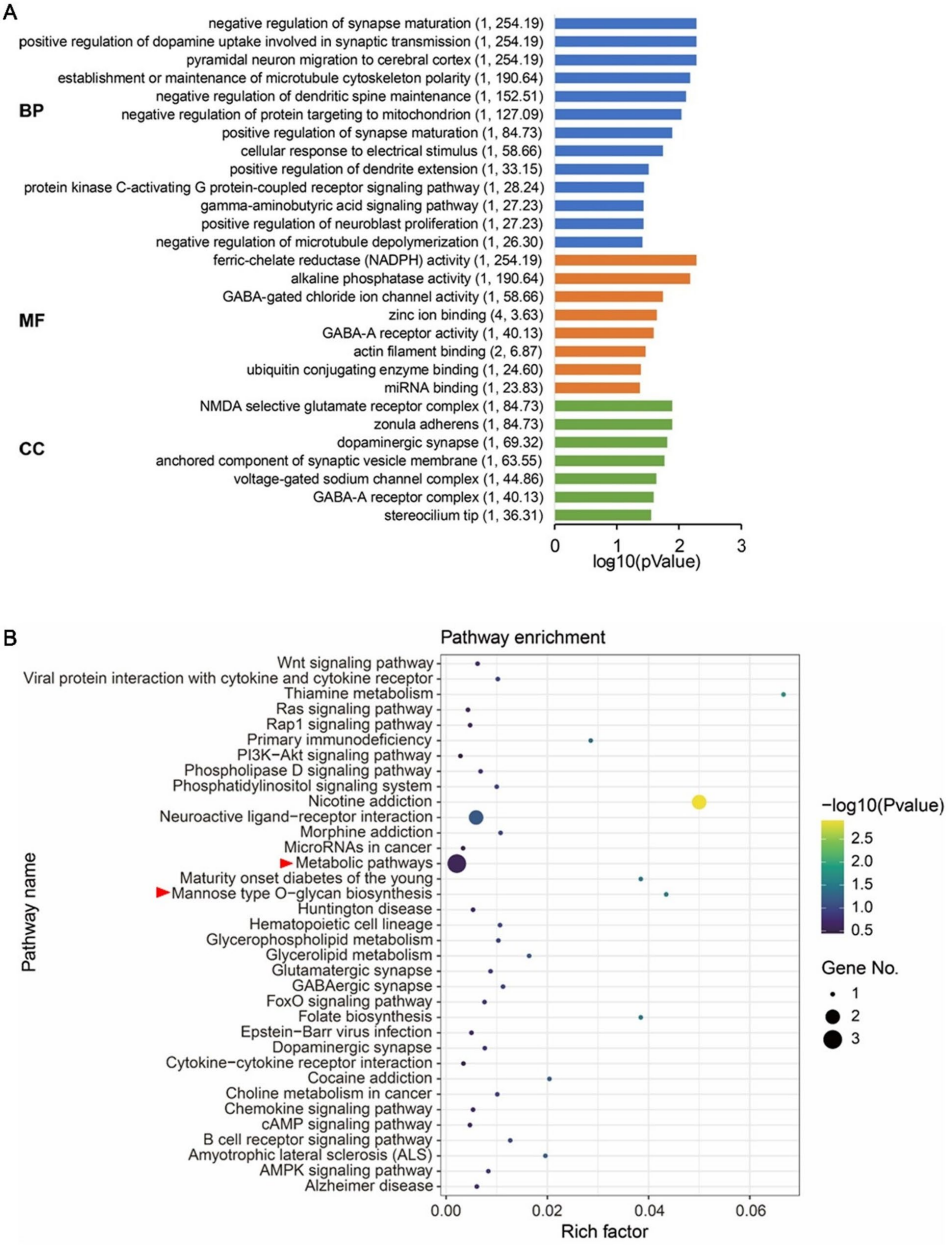
GO and KEGG enrichment analyses of the 29 overlapping DEGs. (**A**) GO analysis showing the top 28 enriched terms classified as BP, MF, and CC. The x-axis was -log10 (*P* value), and the y-axis was the name of the GO terms. (**B**) KEGG analysis showing the top 35 enriched pathways of the DEGs from dataset GSE186542. The x-axis is the rich factor, the size of the dots represents the number of genes, and the gradual colour change ranging from blue (0.5) to yellow (2.5) represents the score of -log10 (*P* value). The y-axis shows the name of the enriched KEGG pathways. Red triangles indicate the functional and signalling pathways in which CPNE6 and B3GAT1 are involved

To further the analysis of these findings, we identified the DEGs via the online dataset GSE186542 (Supplemental Table 7), comparing the mRNA expression profiles of the human degenerative NP tissues to the control samples under the criteria of |log2 FC| > 1 and *p* value < 0.05 (Fig. 4). The volcano plot showed 307 significantly downregulated and 437 significantly upregulated genes (Fig. 4A). Detailed gene expression and distribution status was displayed by heatmap analysis (Fig. 4B). Furthermore, we conducted overlap analysis of the 466 predicted miRNA targets and 744 significant DEGs from the GSE186542 dataset. We identified 27 overlapping genes that may be crucial putative causal genes involved in human NP degeneration. (Fig. 4C and Supplemental Table 8). Additionally, we performed functional and signalling pathway enrichment analyses of these 27 genes based on the GO (Fig. 5A and Supplemental Table 9) and KEGG databases (Fig. 5B and Supplemental Table 10).

### Discovering the DEGs in whole blood of IDD patients via dataset GSE124272

**Fig. 6:**
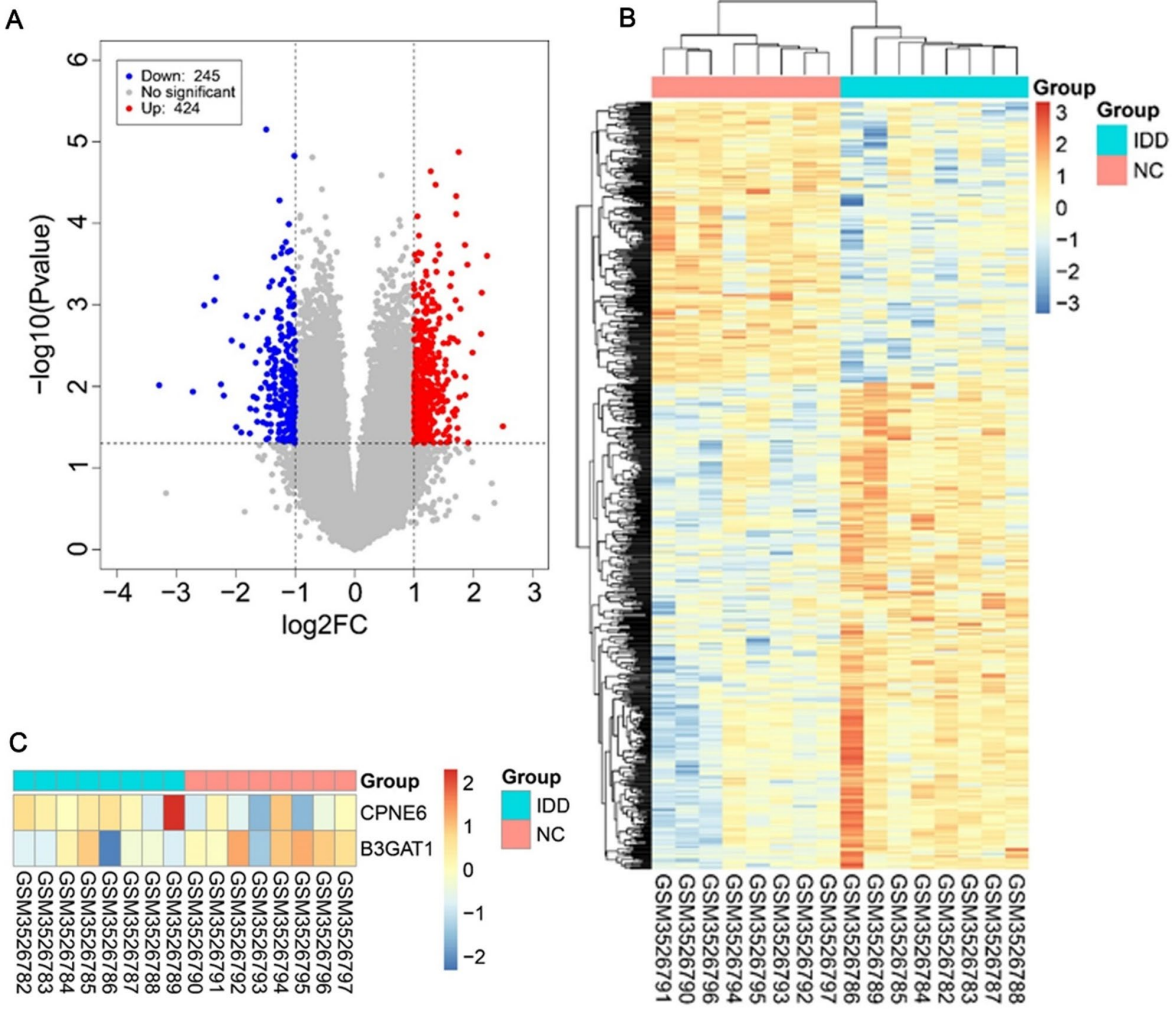
The DEGs from the mRNA expression profile of the whole blood of IDD patients in dataset GSE124272. (**A**) Volcano plot showing 424 significantly upregulated and 245 significantly downregulated mRNA genes. The x-axis is log2 (fold change), and the y-axis is -log10 (*P* value). (**B**) Heatmap of the DEGs from 8 whole blood samples of IDD patients compared to 8 normal controls. Each column represents one tissue sample, and each row represents one gene. The gradual colour change ranging from blue (-3) to red (3) represents the changing expression from downregulation to upregulation. (**C**) Heatmap showing the expression of CPNE6 and B3GAT1 in whole blood of IDD patients

To further investigate the molecular mechanism of IDD progression, we analysed the expression profiles of the whole blood of IDD patients via dataset GSE124272 (Supplemental Table 11). The volcano plot showed 245 significantly downregulated and 424 significantly upregulated mRNAs under the criteria of |log2 FC| > 1 and *p* value < 0.05 (Fig. 6A), and the expression levels of all the DEGs were shown in a heatmap comparing characteristic genes of the whole blood in 8 IDD patients compared to the normal controls (Fig. 6B). Furthermore, we conducted overlap analysis of the 27 crucial genes derived from NP tissues of IDD patients and the DEGs in whole blood of IDD patients and explored two overlapping genes, CPNE6 and B3GAT1 (Fig. 6C and Supplemental Table 12).

### The expression of the two key targets and their corresponding miRNA regulators in IDD patients

**Fig. 7:**
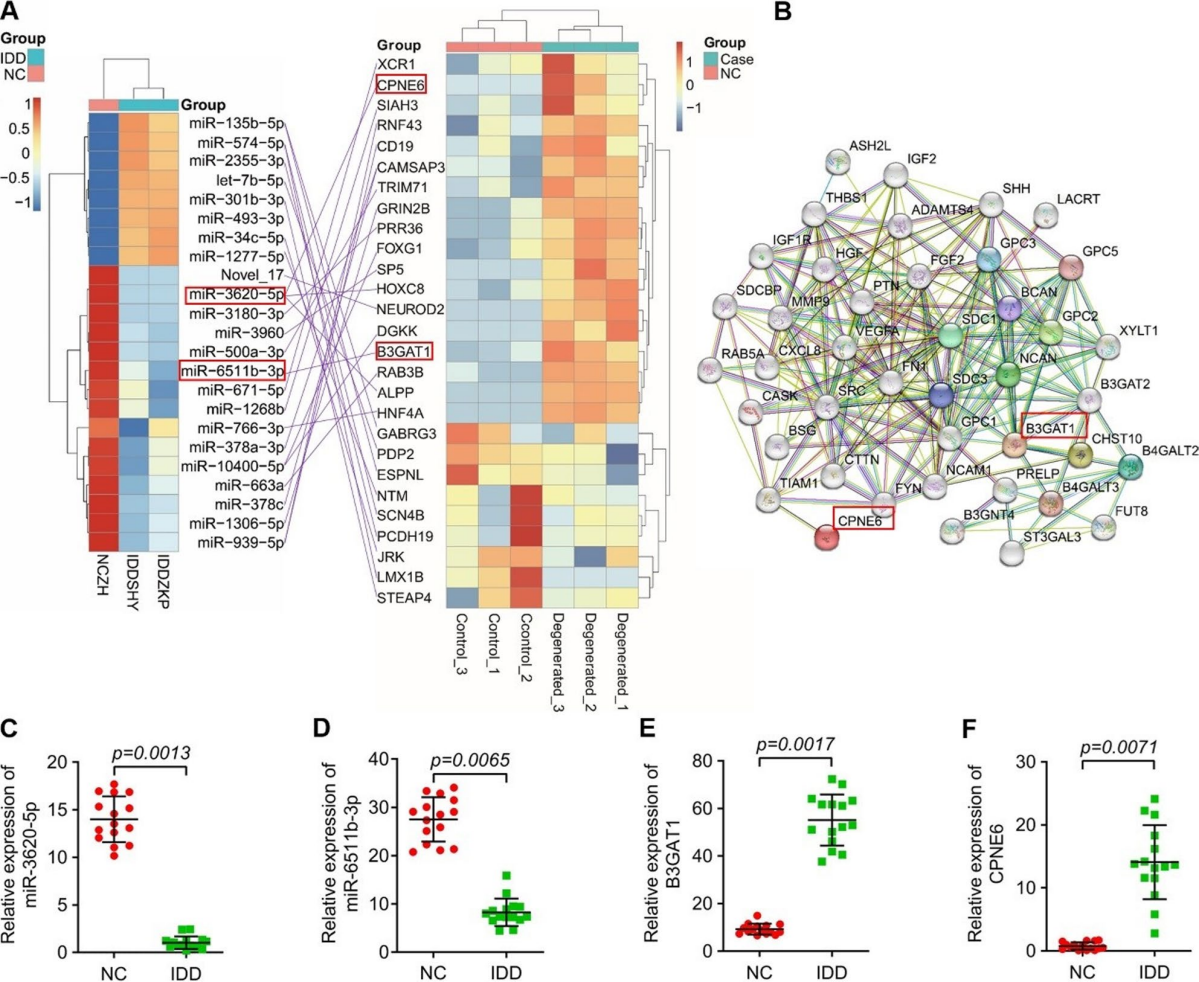
The expression of CPNE6 and B3GAT1 and their corresponding miRNA regulators miR-3620-5p and miR-6511b-3p in NP tissues of IDD patients. (**A**) The 27 overlapping genes and their corresponding miRNA regulators. Blue and red in the heatmap represent downregulation and upregulation, respectively. Red rectangles indicate the CPNE6 and B3GAT1 genes and the corresponding miRNA regulators miR-3620-5p and miR-6511b-3p. (**B**) PPI network analysis by the online STRING database (www.string-db.org) with CPNE6 and B3GAT1 selected as input. (**C**-**F**) The expression levels of miR-3620-5p, miR-6511b-3p, CPNE6 and B3GAT1 in NP tissues of IDD patients. The data are shown as the means ± SDs; *n* = 15

To obtain more information on the molecular regulation underlying IDD progression, we listed the 27 genes from the analysis of NP tissues in IDD patients and their corresponding miRNA regulators (Fig. 7A). The expression of CPNE6 and B3GAT1 was modulated by miR-3620-5p and miR-6511b-3p, respectively (Fig. 7A). PPI network analysis with CPNE6 and B3GAT1 as input showed that they were closely associated with the regulation of a series of proteins, including MMP9, VEGFA, CXCL8, SDC1, SDC3, GPC2 and GPC3 (Fig. 7B). Additionally, we carried out qPCR experiments in NP tissues of 15 IDD patients, and the results showed that both miR-3620-5p (*p* = 0.0013) and miR-6511b-3p (*p* = 0.0065) expression significantly decreased, but CPNE6 (*p* = 0.0071) and B3GAT1 (*p* = 0.0017) expression increased significantly in IDD patients (Fig. 7C and F).

### Both miR-3620-5p and miR-6511b-3p are involved in proliferation and inflammation in ATDC5 cell line

**Fig. 8:**
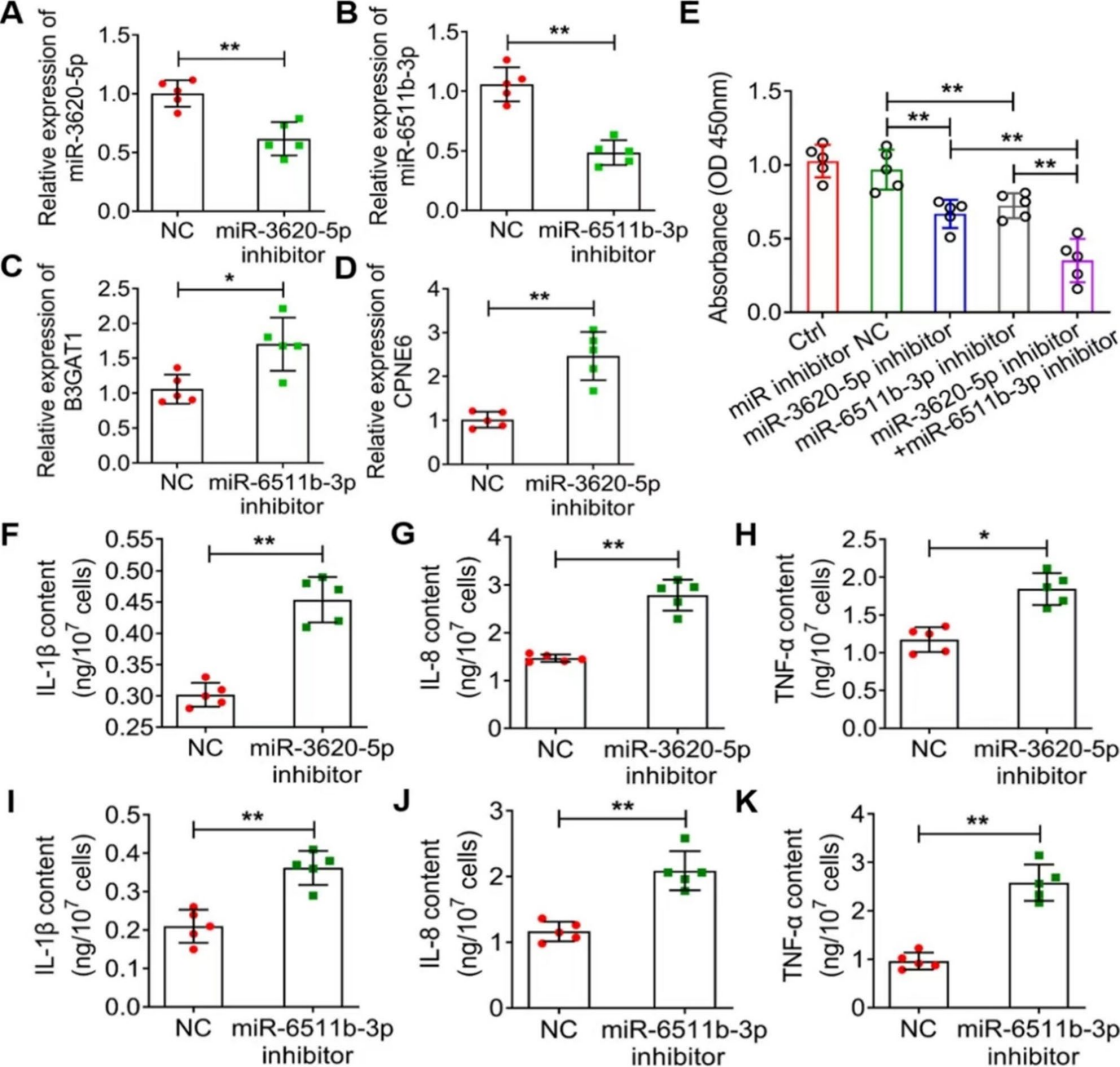
Both miR-3620-5p and miR-6511b-3p are involved in proliferation and inflammation in ATDC5 cell line. (**A**-**D**) The expression levels of miR-3620-5p, miR-6511b-3p, CPNE6 and B3GAT1 in ATDC5 cell transfecting miR-3620-5p and miR-6511b-3p inhibitor. (**E**) CCK8 assay detected the proliferation ATDC5 cell after transfection of miR-3620-5p and miR-6511b-3p inhibitor, respectively. (**F**-**K**) The contents of IL-1β, IL-18 and TNF-α were analysed through ELISA in ATDC5 cell transfecting miR-3620-5p and miR-6511b-3p inhibitor. * *P* < 0.05 and ** *P* < 0.01 vs. the control group (*n* = 5)

To clarify the functional role of miR-3620-5p and miR-6511b-3p in cartilage endplate cells, we succeeded in establishing a stable down expression system of miR-3620-5p and miR-6511b-3p through the miRNA inhibitor transfection of ATDC5 cells (Fig. 8A and D). Then we detected the proliferation of ATDC5 cells via CCK8 assay. As shown in Figs. 8E, compared with the control group, cells proliferation was inhibited after transfection of miR-3620-5p and miR-6511b-3p inhibitor, respectively. In addition, the IL-1β, IL-18 and TNF-α contents in ATDC5 cell transfecting miR-3620-5p and miR-6511b-3p inhibitor were significantly increased compared to those in the control group (Fig. 8F and K). These results suggested that both miR-3620-5p and miR-6511b-3p promoted the cell proliferation and inhibited inflammation in cartilage endplate cells.

## Discussion

IDD is a major contributor to low back, neck and radicular pain, which affects the quality of life of patients and contributes to high economic and social burdens [27, 28]. The pathological features of IDD mainly include NPC senescence and apoptosis, progressive ECM degeneration and degradation, AF fibrosis, and inflammation [29]. Among these, NPC apoptosis and proliferation are revealed to be vital links to IDD [30]. In this study, we performed miRNA sequencing analysis in NP tissues of IDD patients and identified 466 significantly differentially expressed miRNAs, 187 of which were downregulated and 279 or which were upregulated. A series of target genes of these 466 miRNAs were involved in cell migration, axonogenesis, regulation of neuronal synaptic plasticity, positive regulation of cellular component organization, etc., by BP clustering. Moreover, we analysed the mRNA expression profiles of human degradative NP tissues from the online database GSE186542, identifying 744 DEGs that were the main contributors to NP degradation in IDD. Thus, this study confirmed previous findings on IDD formation and progression due to NP pathological changes and deepened the understanding of the molecular regulation underlying IDD.

Currently, the main treatment strategy for IDD includes conservative treatment and surgical treatment based on patients’ symptoms, which can only relieve pain [29]. Even the intervertebral disc fusion strategy that is regarded as the gold standard of surgical treatment for symptomatic IDD patients can cause serious problems in the long run, as it restricts the movement of the adjacent spine body [31]. Accumulating evidence indicates that gene therapy as a biotherapy may provide new directions for IDD treatment, with the possibility of preventing or even reversing IDD. miRNAs are a class of noncoding RNAs (ncRNAs) that generally modulate target gene expression by pairing the miRNA seed region to the complementary sites within target mRNAs and regulating cell apoptosis, cell proliferation, cell autophagy, ECM degeneration or degradation, and the inflammatory response, which play crucial roles in IDD progression [32]. miRNA-targeted therapeutics based on miRNA mimics and molecules targeted at miRNAs (antimiRs) have revealed promise in preclinical development [33]. In this study, we explored 466 differentially expressed miRNAs in NP tissues of IDD patients. After conducting overlap analysis with the miRNA targets and DEGs from dataset GSE186542, we obtained 27 putative causal genes and 23 corresponding miRNA regulators exerting key roles in human NP degradation. These results indicated that high-throughput sequencing analysis was useful to identify putative causal mediators and gain insight into the molecular mechanism of IDD development. Moreover, we explored 245 significantly downregulated and 424 significantly upregulated DEGs in the whole blood of IDD patients from the online dataset GSE124272, within which CPNE6 and B3GAT1 overlapped with the 27 DEGs from degradative NP tissues. Additionally, both CPNE6 and B3GAT1 showed increased expression, but their corresponding miRNA regulators miR-3620-5p and miR-6511b-3p showed decreased expression, in the NP tissues of IDD patients. CPNE6 is a calcium-binding protein that links calcium signals to spine structural plasticity [34]. B3GAT1, as a glucuronyltransferase, is involved in human natural killer-1 carbohydrate (HNK-1) biosynthesis, regulating ECM structures and neurodevelopment [35, 36]. This evidence suggests that CPNE6 and B3GAT1 might be the key regulators affecting ECM and NP structures in intervertebral discs and that abnormal expression of these genes and their miRNA regulators might result in IDD progression. Moreover, results in this study indicated that both miR-3620-5p and miR-6511b-3p promoted the cell proliferation and inhibited inflammation in cartilage endplate cells, which might clarify that miR-3620-5p and miR-6511b-3p might direct inflammation-mediated ECM and NP remodeling during IDD progression.

In conclusion, this study utilized miRNA sequencing of NP tissues from IDD patients and bioinformatics analysis of the mRNA expression profiles of human degenerative NP from the online database GSE186542 and whole blood of IDD patients from the online database GSE124272 to discover miRNAs, identify their targets and further infer their functions in IDD formation and progression. Ultimately, we suggest that miR-3620-5p and miR-6511b-3p might play key roles in maintaining ECM and NP structures by mediating the expression of CPNE6 and B3GAT1. However, the detailed regulatory relationships between the potential miRNA‒mRNA regulatory axis are needed more comprehensive experimental data. And CPNE6 and B3GAT1 involvement in inflammation response and cell activity regulation are lacking evidences. Thus, more investigations are urgently needed in the future to verify their roles in IDD. This study accumulated evidence for ncRNA-based therapy application for IDD diagnosis, prevention, and treatment. However, some certain limitations should be acknowledged. One significant limitation is the sample size. A relatively small sample size might have restricted the statistical power and the generalizability of the results. With a limited number of samples, it becomes more difficult to detect subtle effects or to draw definitive conclusions about the broader population. For example, in the analysis of miRNA-mRNA interactions, a larger sample size could potentially uncover rarer but biologically relevant relationships. To address these limitations in future research, several strategies could be implemented. Firstly, increasing the sample size is crucial. This could involve collaborating with multiple research centers or institutions to pool samples, thereby enhancing the statistical robustness of the analysis. A larger sample size would allow for more detailed subgroup analyses, such as exploring differences between genders, age groups, or different disease subtypes. Secondly, broadening the selection criteria in a more systematic way could improve the generalizability. Instead of focusing on a very specific subset of the population, a more inclusive approach could be taken, followed by post - hoc stratification and analysis to understand the heterogeneity within the sample. Additionally, prospective cohort studies could be designed, where a more diverse range of participants are enrolled and followed over time. This would enable the capture of a more comprehensive set of variables and potential confounding factors, providing a more accurate picture of the relationship between miRNAs, mRNAs, and the associated biological processes in IDD.

## Supplementary Information

Supplementary Material 1.

Supplementary Material 2.

Supplementary Material 3.

Supplementary Material 4.

Supplementary Material 5.

Supplementary Material 6.

Supplementary Material 7.

Supplementary Material 8.

Supplementary Material 9.

Supplementary Material 10.

Supplementary Material 11.

Supplementary Material 12.

## Data Availability

The datasets generated and/or analysed during the current study are available in the SRA repository (SRR30615492, [https://www.ncbi.nlm.nih.gov/sra/?term=SRR30615492](https:/www.ncbi.nlm.nih.gov/sra/?term=SRR30615492) ) and the GEO repository (GSE186542, [www.ncbi.nlm.nih.gov/geo/query/acc.cgi? acc=GSE186542](http:/www.ncbi.nlm.nih.gov/geo/query/acc.cgi? acc=GSE186542) ; GSE124272, www.ncbi.nlm.nih.gov/geo/query/acc.cgi? acc=GSE124272).

## Declarations

### Running title

Potential miRNA‒mRNA Regulatory Axis in intervertebral disc degeneration.

## References

[1] Vergroesen PP, Kingma I, Emanuel KS, Hoogendoorn RJ, Welting TJ, van Royen BJ, van Dieen JH, Smit TH. Mechanics and biology in intervertebral disc degeneration: a vicious circle. Osteoarthr Cartil. 2015;23(7):1057–70. 10.1016/j.joca.2015.03.028 25827971

[2] Kirnaz S, Capadona C, Wong T, Goldberg JL, Medary B, Sommer F, McGrath LB Jr, Hartl R. Fundamentals of Intervertebral Disc Degeneration. World Neurosurg. 2022;157:264–73. 34929784 10.1016/j.wneu.2021.09.066

[3] Cheng F, Yang H, Cheng Y, Liu Y, Hai Y, Zhang Y. The role of oxidative stress in intervertebral disc cellular senescence. Front Endocrinol. 2022;13:1038171. 10.3389/fendo.2022.1038171 PMC9763277 36561567

[4] Krut Z, Pelled G, Gazit D, Gazit Z. Stem Cells and Exosomes: New Therapies for Intervertebral Disc Degeneration. Cells. 2021;10(9):2241. 34571890 10.3390/cells10092241 PMC8471333

[5] Chen J, Yang X, Feng Y, Li Q, Ma J, Wang L, Quan Z. Targeting Ferroptosis Holds Potential for Intervertebral Disc Degeneration Therapy. Cells. 2022;11(21):3508. 36359904 10.3390/cells11213508 PMC9653619

[6] Adams MA, Roughley PJ. What is intervertebral disc degeneration, and what causes it? Spine. 2006;31(18):2151–61. 16915105 10.1097/01.brs.0000231761.73859.2c

[7] Smith LJ, Fazzalari NL. Regional variations in the density and arrangement of elastic fibres in the anulus fibrosus of the human lumbar disc. J Anat. 2006;209(3):359–67. 16928204 10.1111/j.1469-7580.2006.00610.x PMC2100325

[8] Liebscher T, Haefeli M, Wuertz K, Nerlich AG, Boos N. Age-related variation in cell density of human lumbar intervertebral disc. Spine. 2011;36(2):153–9. 20671592 10.1097/BRS.0b013e3181cd588c

[9] Gan Y, Li P, Wang L, Mo X, Song L, Xu Y, Zhao C, Ouyang B, Tu B, Luo L, et al. An interpenetrating network-strengthened and toughened hydrogel that supports cell-based nucleus pulposus regeneration. Biomaterials. 2017;136:12–28. 28505597 10.1016/j.biomaterials.2017.05.017

[10] Wang X, He Y, Mackowiak B, Gao B. MicroRNAs as regulators, biomarkers and therapeutic targets in liver diseases. Gut. 2021;70(4):784–95. 33127832 10.1136/gutjnl-2020-322526

[11] Yan C, Chen J, Wang C, Yuan M, Kang Y, Wu Z, Li W, Zhang G, Machens HG, Rinkevich Y, et al. Milk exosomes-mediated miR-31-5p delivery accelerates diabetic wound healing through promoting angiogenesis. Drug Delivery. 2022;29(1):214–28. 34985397 10.1080/10717544.2021.2023699 PMC8741248

[12] Zhao Z, Sun W, Guo Z, Zhang J, Yu H, Liu B. Mechanisms of lncRNA/microRNA interactions in angiogenesis. Life Sci. 2020;254:116900. 31786194 10.1016/j.lfs.2019.116900

[13] Ali Syeda Z, Langden SSS, Munkhzul C, Lee M, Song SJ. Regulatory Mechanism of MicroRNA Expression in Cancer. Int J Mol Sci. 2020;21(5):1723. 32138313 10.3390/ijms21051723 PMC7084905

[14] Huang Y. The novel regulatory role of lncRNA-miRNA-mRNA axis in cardiovascular diseases. J Cell Mol Med. 2018;22(12):5768–75. 30188595 10.1111/jcmm.13866 PMC6237607

[15] Wang C, Cui L, Gu Q, Guo S, Zhu B, Liu X, Li Y, Liu X, Wang D, Li S. The Mechanism and Function of miRNA in Intervertebral Disc Degeneration. Orthop Surg. 2022;14(3):463–71. 35142050 10.1111/os.13204 PMC8926997

[16] Cazzanelli P, Wuertz-Kozak K. MicroRNAs in Intervertebral Disc Degeneration, Apoptosis, Inflammation, and Mechanobiology. Int J Mol Sci. 2020;21(10):3601. 32443722 10.3390/ijms21103601 PMC7279351

[17] Zhao Y, Li A. miR-19b-3p relieves intervertebral disc degeneration through modulating PTEN/PI3K/Akt/mTOR signaling pathway. Aging. 2021;13(18):22459–73. 34554926 10.18632/aging.203553 PMC8507280

[18] Xu J, Xie G, Yang W, Wang W, Zuo Z, Wang W. Platelet-rich plasma attenuates intervertebral disc degeneration via delivering miR-141-3p-containing exosomes. Cell Cycle. 2021;20(15):1487–99. 34229586 10.1080/15384101.2021.1949839 PMC8354670

[19] Li Z, Wu Y, Tan G, Xu Z, Xue H. Exosomes and exosomal miRNAs: A new therapy for intervertebral disc degeneration. Front Pharmacol. 2022;13:992476. 36160436 10.3389/fphar.2022.992476 PMC9492865

[20] Xing H, Zhang Z, Mao Q, Wang C, Zhou Y, Zhou X, Ying L, Xu H, Hu S, Zhang N. Injectable exosome-functionalized extracellular matrix hydrogel for metabolism balance and pyroptosis regulation in intervertebral disc degeneration. J Nanobiotechnol. 2021;19(1):264. 10.1186/s12951-021-00991-5 PMC8419940 34488795

[21] Urrutia J, Besa P, Campos M, Cikutovic P, Cabezon M, Molina M, Juan Pablo Cruz. The Pfirrmann classification of lumbar intervertebral disc degeneration: an independent inter- and intra-observer agreement assessment. Eur Spine J. 2016;25(9):2728–33. 26879918 10.1007/s00586-016-4438-z

[22] Pratibha Potla SA, Ali M, Kapoor. A bioinformatics approach to microRNA-sequencing analysis. Osteoarthr Cartil Open. 2020;3(1):100131. 36475076 10.1016/j.ocarto.2020.100131 PMC9718162

[23] Shirley Tam M-S, Tsao, John D, McPherson. Optimization of miRNA-seq data preprocessing. Brief Bioinform. 2015;16(6):950–63. 25888698 10.1093/bib/bbv019 PMC4652620

[24] Weijun Liu X, Wang. Prediction of functional microRNA targets by integrative modeling of microRNA binding and target expression data. Genome Biol. 2019;20(1):18. 30670076 10.1186/s13059-019-1629-z PMC6341724

[25] Hung K-S, Hsiao C-C, Pai T-W, Hu C-H, Tzou W-S, Wang W-D. Yet-Ran Chen. Functional enrichment analysis based on long noncoding RNA associations. BMC Syst Biol. 2018;12(Suppl 4):45. 29745842 10.1186/s12918-018-0571-0 PMC5998891

[26] Xu R, Wei Y, Yin X, Shi B, Li J. miR-20a suppresses chondrogenic differentiation of ATDC5 cells by regulating Atg7. Sci Rep. 2019;9(1):9243. 31239522 10.1038/s41598-019-45502-7 PMC6592888

[27] Genereaux D, van Karnebeek CD, Birch PH. Costs of caring for children with an intellectual developmental disorder. Disabil health J. 2015;8(4):646–51. 25991418 10.1016/j.dhjo.2015.03.011

[28] Akobirshoev I, Mitra M, Parish SL, Moore Simas TA, Dembo R, Ncube CN. Racial and ethnic disparities in birth outcomes and labour and delivery-related charges among women with intellectual and developmental disabilities. J Intellect Disabil research: JIDR. 2019;63(4):313–26. 30576027 10.1111/jir.12577 PMC7271252

[29] Xin J, Wang Y, Zheng Z, Wang S, Na S, Zhang S. Treatment of Intervertebral Disc Degeneration. Orthop Surg. 2022;14(7):1271–80. 35486489 10.1111/os.13254 PMC9251272

[30] Feng Y, Egan B, Wang J. Genetic Factors in Intervertebral Disc Degeneration. Genes Dis. 2016;3(3):178–85. 27617275 10.1016/j.gendis.2016.04.005 PMC5016799

[31] Rajaee SS, Bae HW, Kanim LE, Delamarter RB. Spinal fusion in the United States: analysis of trends from 1998 to 2008. Spine. 2012;37(1):67–76. 21311399 10.1097/BRS.0b013e31820cccfb

[32] Agarwal V, Bell GW, Nam JW, Bartel DP. Predicting effective microRNA target sites in mammalian mRNAs. eLife. 2015;4:e05005. 26267216 10.7554/eLife.05005 PMC4532895

[33] Rupaimoole R, Slack FJ. MicroRNA therapeutics: towards a new era for the management of cancer and other diseases. Nat Rev Drug Discovery. 2017;16(3):203–22. 28209991 10.1038/nrd.2016.246

[34] Reinhard JR, Kriz A, Galic M, Angliker N, Rajalu M, Vogt KE, Ruegg MA. The calcium sensor Copine-6 regulates spine structural plasticity and learning and memory. Nat Commun. 2016;7:11613. 27194588 10.1038/ncomms11613 PMC4874034

[35] Kahler AK, Djurovic S, Rimol LM, Brown AA, Athanasiu L, Jonsson EG, Hansen T, Gustafsson O, Hall H, Giegling I, et al. Candidate gene analysis of the human natural killer-1 carbohydrate pathway and perineuronal nets in schizophrenia: B3GAT2 is associated with disease risk and cortical surface area. Biol Psychiatry. 2011;69(1):90–6. 20950796 10.1016/j.biopsych.2010.07.035

[36] Jeffries AR, Mungall AJ, Dawson E, Halls K, Langford CF, Murray RM, Dunham I, Powell JF. beta-1,3-Glucuronyltransferase-1 gene implicated as a candidate for a schizophrenia-like psychosis through molecular analysis of a balanced translocation. Mol Psychiatry. 2003;8(7):654–63. 12874601 10.1038/sj.mp.4001382

